# Inflammatory pseudotumor of kidney: a challenging diagnostic entity

**DOI:** 10.1590/S1677-5538.IBJU.2017.0063

**Published:** 2018

**Authors:** Anudeep Mukkamala, Robin M. Elliott, Nicholas Fulton, Vikas Gulani, Lee E. Ponsky, Riccardo Autorino

**Affiliations:** 1 Department of Urology, UH Case Medical Center, Cleveland, Ohio, USA; 2 Department of Pathology, University Hospitals Case Medical Center, Cleveland, Ohio, USA; 3 Department of Radiology, University Hospitals Case Medical Center, Cleveland, Ohio, USA

## DESCRIPTION OF CASE

**Figure 1:**
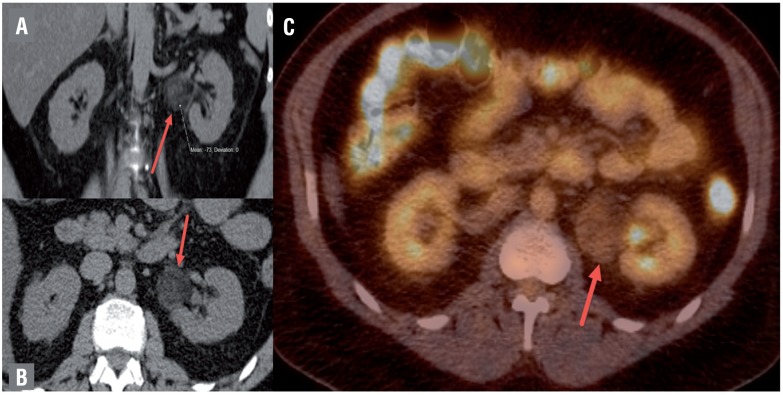
CT Images. a) Coronal non-contrast CT demonstrating a peri-renal mass with heterogeneous attenuation. Hounsfield units measured at −73 indicating fat in mass. b) Axial non-contrast CT again demonstrating left perirenal mass with heterogeneous attenuation arising from retroperitoneum. c) Axial PET/CT shows mild increased FDG uptake with SUV (standardized uptake value) measurements of up to 2.8, which could represent malignancy or inflammatory process.

**Figure 2:**
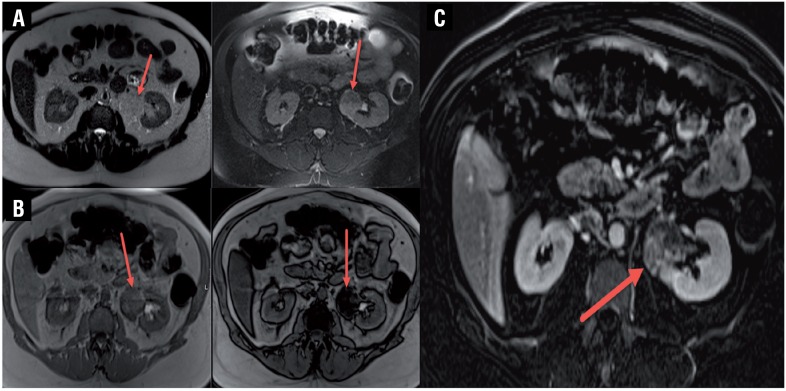
MRI Images. a) Axial MRI, HASTE (T2-weighted) (left) and HASTE with fat-saturation (right) demonstrates the mass. b) Axial MRI, in phase (left) and opposed phase (right), c) Axial MRI, post-contrast subtracted image shows areas of enhancement in mass.

A 54-year-old otherwise healthy Caucasian male was referred to us for further management of an enlarging left heterogeneous peri-renal mass. The patient had been initially followed with serial imaging by his primary care physician, who had been monitoring the mass since it was first detected 2 years earlier, when it was 2.9cm in size. Most recent CT scan showed a 4.7 cm mass, located posteriorly, near the renal hilum, apparently peri-renal (arising from retroperitoneum). Axial PET/CT showed mild increased FDG uptake with standardized uptake value measurements of up to 2.8, which could represent malignancy or inflammatory process (Figure-1). It was decided to obtain a CT guided biopsy of the mass, which showed adipose tissue with cytologic atypia and inflammation. A MRI was also performed but this did not allow a definitive diagnosis, with findings compatible with a range of entity, from angiomyolipoma to liposarcoma to renal cell carcinoma (1–4) (Figure-2). Therefore, surgical options were discussed with patient. Since the mass had doubled in size in about 2 years, it was decided to proceed with laparoscopic removal of the mass. Intraoperatively, decision was made to convert to laparoscopic radical nephrectomy due to inability to safely excise the mass from the major renal vessels.

**Figure 3:**
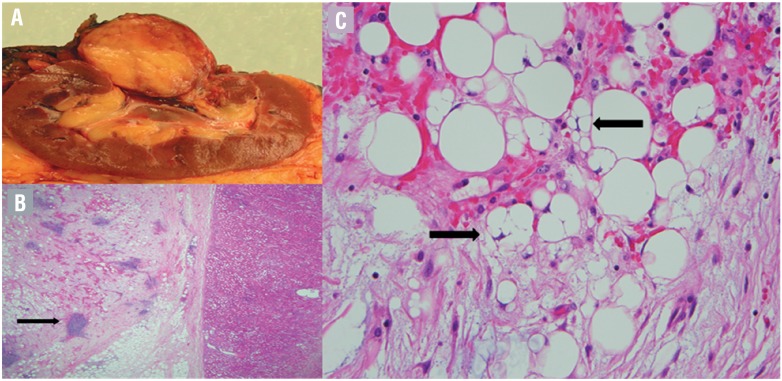
Histopathology. a) Gross: bivalved kidney with well circumscribed tumor near hilum. b) Microscopic, 20x: sclerotic adipose tissue with foci of chronic inflammation (arrow), c) Microscopic, 400x: multivacuolated pseudolipoblasts (arrows).

Gross examination of the surgical specimen revealed a well-circumscribed mass displaying fibrous and fatty cut surfaces. Microscopic examination revealed a proliferation of spindle cells with features of reactive myofibroblasts and fibroblasts interspersed with lobules of adipocytes, many of which contained multiple vacuoles, characteristic of pseudolipoblasts. Foamy macrophages and infiltrates of lymphocytes and plasma cells were present throughout the tumor. Based on the morphologic features, the differential diagnosis included well-differentiated liposarcoma, inflammatory myofibroblastic tumor, angiomyolipoma, an IgG4-related sclerosing lesion, and inflammatory pseudotumor. Immunohistochemical stains showed that the myofibrobasts were positive for CD34, vimentin, and smooth muscle actin and were negative for melan-A, HMB-45, ALK, and bcl-2. IgG4 staining did not reveal an excessive number of IgG4-positive plasma cells. Fluorescent in situ hybridization revealed no amplification of the MDM2 gene locus, ruling out the possibility of well-differentiated liposarcoma. The overall findings were most consistent with inflammatory pseudotumor (Figure-3). Postoperative course was uneventful and patient is disease-free after a follow-up of 12 months.

This type of lesion is rarely encountered (5–8), but its variable and nonspecific features on imaging makes a preoperative clinical diagnosis quite challenging. Differential diagnoses include malignant tumors such as renal cell carcinoma, sarcomatoid renal cell carcinoma, inflammatory fibrosarcoma, malignant fibrous histiocytoma, low grade neurogenic tumor, myxoid leiomyosarcoma and non-malignant tumors such as angiomyolipoma, xanthogranuloma pyelonephritis and plasma cell granuloma. It is difficult to make a preoperative diagnosis because symptoms and imaging findings are not specific. Renal biopsy in this scenario is likely failing a definitive diagnosis. It is therefore appropriate to presume the given renal mass to be a renal cell carcinoma and to perform nephrectomy (radical or partial) (5–8).

In conclusion, inflammatory pseudotumor of the kidney represents an extremely rare neoplasm of uncertain biological potential, which the clinician should keep in mind in the differential diagnosis of an enlarging renal mass with non specific features on diagnostic imaging and inconclusive biopsy results.
